# Development of uMUC-1 Targeted NEMO Particles with pH-Activatable MRI Signals for Enhanced Detection of Malignant Breast Cancer Cells

**DOI:** 10.1021/acsabm.5c00365

**Published:** 2025-05-01

**Authors:** Dhruvi M. Panchal, Alexia R. Gorman, Celia Martinez de la Torre, Barrick M. Silverman, Anthony J. Scalzo, Hunter T. Snoderly, Benoit Driesschaert, Margaret F. Bennewitz

**Affiliations:** † Department of Chemical and Biomedical Engineering, Benjamin M. Statler College of Engineering and Mineral Resources, West Virginia University, Morgantown, West Virginia 26506, United States; ‡ Department of Biology, Eberly College of Arts and Sciences, West Virginia University, Morgantown, West Virginia 26506, United States; § Department of Pharmaceutical Sciences, School of Pharmacy, West Virginia University, Morgantown, West Virginia 26506, United States; ∥ In Vivo Multifunctional Magnetic Resonance Center, School of Medicine, West Virginia University, Morgantown, West Virginia 26506, United States; ⊥ Eugene Bennett Department of Chemistry, Eberly College of Arts and Sciences, West Virginia University, Morgantown, West Virginia 26506, United States

**Keywords:** magnetic resonance imaging, manganese oxide, nanoparticles, uMUC-1, breast cancer

## Abstract

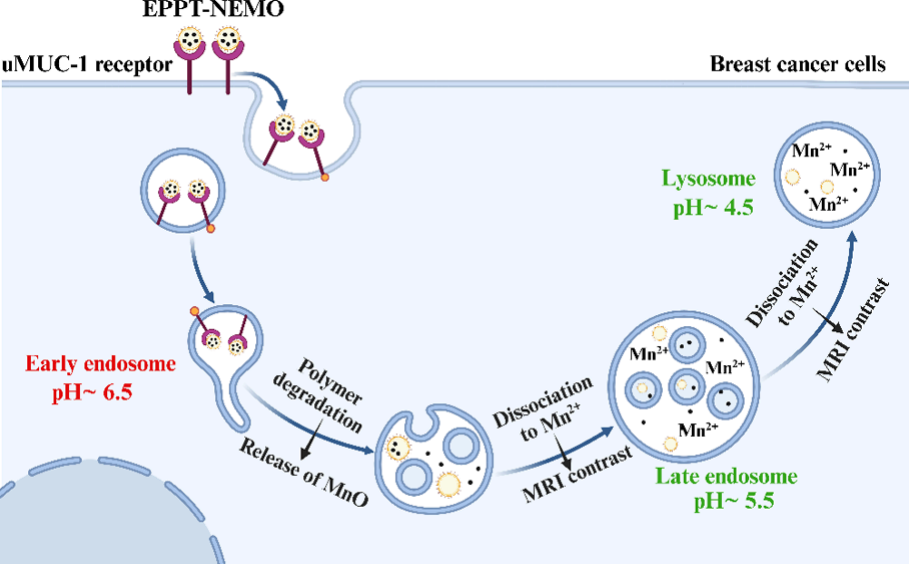

Magnetic resonance imaging (MRI) detects more breast cancers than mammography due to its superior soft tissue contrast; however, it still misdiagnoses 40% of benign tumors as malignant due to clinically used nonspecific contrast agents (e.g., gadolinium chelates). To overcome this limitation, we developed receptor-targeted, pH-sensitive Nano-Encapsulated Manganese Oxide (NEMO) particles as an alternative *T*_1_-weighted MRI contrast agent. A breast cancer targeting peptide, EPPT, against underglycosylated mucin-1, promotes preferential endocytosis of NEMO particles by malignant cells and specific activation of the MRI signal inside low pH endosomes/lysosomes. In just 30 min, EPPT-NEMO particles produced rapid and robust *T*_1_-weighted MRI contrast inside T47D breast cancer cells that reached ∼276% signal enhancement, which was significantly brighter than MCF10A benign control cells (∼57% enhancement). Mn cellular content further confirmed peptide targeting specificity, while confocal microscopy showed the colocalization of EPPT-NEMO particles with endosomes and lysosomes. EPPT-NEMO particles show promise as alternative *T*_1_-weighted MRI contrast agents, producing significantly brighter signals in breast cancer cells compared to benign cells within clinically relevant timeframes. These advancements in targeted MRI contrast agents could lead to improved accuracy in breast cancer diagnosis and ultimately to better patient outcomes.

## Introduction

Breast cancer is the second most common cancer worldwide and is the fourth greatest cause of cancer-related death in the world.^1^ The diagnostic stage and molecular subtype greatly impact the patient's prognosis. The 5-year survival rate decreases from 99% for localized disease to 30% with distant metastasis.^2^ The earlier the tumor is diagnosed, the sooner treatment can be applied to increase survival. Currently, mammography is the primary screening tool that aims to promote early detection; however, it misses 20% of breast cancers, often in young women with dense breasts.^3,4^ Moreover, 50% of healthy women will experience a false positive diagnosis over 10 years of annual screening, wherein a benign mass is incorrectly diagnosed as malignant.^3^ False positives cause anxiety, distress, and increased medical expenses due to additional testing.^5^

As an adjunct to mammography, magnetic resonance imaging (MRI) is used for high-risk women who have a strong family history of breast cancer or dense breast tissue. Compared to mammography, MRI has superior contrast to visualize structural differences within soft tissue and detect more breast cancers.^2,3^ MRI also produces 3D images of the tumor and does not require X-ray radiation or breast compression. Despite increased sensitivity, MRI still misses 5–15% of breast cancers^6^ and misdiagnoses ∼40% of benign masses as breast cancers^7^ due to the intravenous contrast agent currently used (e.g., gadolinium chelates). Clinically used gadolinium (Gd) chelates are always “ON”, creating a signal wherever the agent accumulates in the body. Moreover, Gd chelates lack specificity^8^ and enter highly vascularized tissues of benign and malignant tumors.

In light of these challenges, there is an unmet need for alternative MRI contrast agents that offer enhanced specificity for malignant tumors and minimize false positive diagnoses. Manganese oxide (MnO) nanocrystals are pH-sensitive contrast agents that can switch from an “OFF” state to an “ON” state in acidic environments, generating bright *T*_1_ MRI contrast.^9−11^ At the physiological pH of the bloodstream, the particles are intact and do not cause MRI contrast. Within the tumor extracellular space, MnO is minimally dissociated due to the slightly acidic environment (pH 6.5–6.9) from lactic acid buildup. However, after cancer cell uptake into the acidic endosomes/lysosomes (pH ∼ 5), MnO robustly dissociates into Mn^2+^, which coordinates with water molecules to produce a positive MRI signal, turning “ON” MRI contrast.^10,12−16^ The combination of MnO’s pH-switchability with specific targeting of breast cancer could combat the lack of specificity that the current Gd-based contrast agents demonstrate.

Breast cancers can be grouped into four basic subtypes depending on the expression of the estrogen receptor (ER), progesterone receptor (PR), and human epidermal growth factor receptor 2 (HER2): luminal A (ER+/PR+/HER2-), luminal B (ER+/PR+/HER2+), HER2 positive (ER-/PR-/HER2+), and basal-like or triple negative (ER-/PR-/HER2-).^17^ Despite luminal A being the most prevalent (68% of cases),^2^ it is poorly researched relative to other subtypes. One reason might be due to the estrogen dependency of luminal A cancers,^18,19^ rendering it difficult to be grown in animal models. The biomarkers for subtyping (HER2, ER, PR) and others associated with breast cancer (e.g., folate receptor) could be used to target MnO nanoparticles; however, these biomarkers are either in the cell nucleus^20,21^ or expressed at relatively low rates.^22,23^

Mucin-1 (MUC-1) is a promising target for enhanced cancer detection, as it is overexpressed in >90% of breast cancers and is prevalent in colorectal, ovarian, pancreatic, prostate, and lung carcinomas.^24^ The extracellular portion of mucins consists of around 20 to 120 or more repetitions of a 20 amino acid residue sequence (HGVTSAPDTRPAPGSTAPPA) containing five O-linked glycosylation sites. In normal tissue, MUC-1 is a heavily glycosylated transmembrane protein, where carbohydrates form 50–90% of its molecular weight, whereas, in cancerous tissue, MUC-1 loses its polarity and is underglycosylated (i.e., uMUC-1).^24−26^ The MUC-1-derived peptide sequences, including RPAPGS, PPAHGVT, and PDTRP, are the most frequent minimal epitopes being identified.^27^ The PDTRP motif is the most widespread epitope to facilitate binding with targeting peptides.^28,29^ On normal and benign cells, PDTRP is hidden by MUC-1’s dense hyperglycosylation, but is revealed on cancer cells due to the truncated glycosylation of uMUC-1.^30−32^ The uMUC-1 targeting peptide, EPPT, is derived from the CDR3 *V*_h_ region of the ASM2 monoclonal antibody against human epithelial cancer and has a high affinity for uMUC-1 (*K*_d_ = 20 μM).^24^ uMUC-1 targeting peptides have enhanced cancer therapy and imaging.^24,33−37^ Thus, uMUC-1 targeting is attractive to increase MRI contrast agent specificity in breast cancer cells.

In this study, nano-, encapsulated manganese oxide (NEMO) particles were innovatively combined with a uMUC-1 targeting peptide for enhanced detection of breast cancer. As uMUC-1 epitopes are only revealed in malignancy, we hypothesized that mammary carcinoma cells would preferentially endocytose uMUC-1-targeted EPPT-NEMO particles compared with benign cells, generating specific MRI signals inside low pH endosomes and lysosomes of breast cancer cells. The cellular MRI signal after EPPT-NEMO particle labeling at different time points over 1 h was assessed to determine the optimal time for robust *T*_1_ MRI signal activation. MRI studies were complemented by analysis of internalized total Mn content per cell, particle localization within early endosomes, late endosomes, and lysosomes of cells, and cytotoxicity. The *T*_1_-weighted MRI contrast of EPPT-NEMO in T47D breast cancer cells significantly peaked to ∼276% signal enhancement in just 30 min, which was significantly greater than controls (T47D cells labeled with scrambled peptide NEMO or MCF10A benign cells labeled with EPPT-NEMO). From 30 to 45 min, EPPT-NEMO particles continued to generate significantly greater *T*_1_-weighted MRI contrast and intracellular Mn content in malignant T47D cells versus those in benign MCF10A controls. EPPT-NEMO particles are a promising alternative *T*_1_-weighted MRI contrast agent that produces a significantly brighter signal in breast cancer cells after just 30 min of incubation, which supports clinically relevant timeframes for signal activation.

## Experimental Section

### Materials

Manganese(II) acetylacetonate (Mn(II)(acac)_2_) (technical grade, ≥97%), oleylamine (technical grade, 70%), poly(vinyl alcohol) (PVA), cell proliferation reagent WST-1, and human IgG were purchased from Sigma-Aldrich. Dibenzyl ether (≥99%, Acros Organics), hexane (≥98.5%, Macron Fine Chemicals), dichloromethane (99.5%, BDH Chemicals), copper(II) acetate monohydrate (98%, extra pure, Acros Organics), tris((1-benzyl-4-triazolyl)methyl)amine (TBTA) (97+%, Alfa Aesar), sodium ascorbate (99%, Alfa Aesar), manganese(II) chloride tetrahydrate (98–101% ACS), Dulbecco’s phosphate-buffered saline (PBS), RPMI-1640 media, fetal bovine serum (FBS), penicillin-streptomycin (Corning), formaldehyde (16% Ultra-Pure EM grade, Polysciences Inc.), sodium citrate dihydrate (BDH Chemicals), agarose, and citric acid were bought from VWR (VWR Chemicals). MEGM Mammary Epithelial Cell Growth Medium BulletKit was obtained from Lonza. Carboxylic acid-terminated, 50:50 poly(D,l-lactide-*co*-glycolide) (PLGA) (viscosity: 0.55–0.75 dL/g) was obtained from LACTEL Absorbable Polymers. PLGA (35 kDa)–PEG (2 kDa)–alkyne (ALK) was obtained from Nanosoft Polymers. Hydrochloric acid (HCl) TraceMetal grade was acquired from Fisher Scientific. Cy5 fluorophore was obtained from APExBIO. Pierce quantitative fluorometric peptide assay, Hoechst 33342, and CellLight Fluorescent Protein Labeling BacMam 2.0 kits for early endosome (RFP, red-fluorescence tag), late endosome, and lysosome (GFP, green-fluorescence tag) were purchased from Thermo Fisher. Antibodies CD227 and SM3 were obtained from BioLegend and Santa Cruz Technologies, respectively. Ethanol (Decon Laboratories) was obtained internally from West Virginia University’s Environmental Health and Safety Services.

### Cell Culture

MCF10A human nontumorigenic epithelial cells (benign fibrocystic disease) and T47D human luminal A breast cancer cells were a kind gift from Dr. Elena Pugacheva. All cells were cultured in a humidified incubator at 37 °C with 5% CO_2_. MCF10A cells were cultured in MEGM growth medium supplemented with 10% FBS, 1% penicillin-streptomycin, bovine pituitary extract, hydrocortisone, human epidermal growth factor, insulin, and gentamicin/amphotericin-B. T47D were cultured in RPMI-1640 medium supplemented with 10% FBS, 1% penicillin-streptomycin, 10 mM HEPES, 1 mM sodium pyruvate, and 4500 mg/L glucose.

### Synthesis of MnO Cores and NEMO Particles

MnO cores were synthesized and characterized according to previously optimized methods by thermal decomposition of Mn(II)(acac)_2_ (1.51 g) with oleylamine (10 mL) and dibenzyl ether (50 mL) at 280 °C for 30 min with a ramping rate of 10 °C/min.^38^ Using a single oil-in-water emulsion, 50 mg MnO cores were encapsulated within 100 mg of PLGA with 2.5% w/w PLGA–PEG-ALK with dichloromethane as the organic solvent and 10% PVA as the stabilizer; blank particles were synthesized with no MnO cores.^39^ For confocal microscopy, Cy5 dye (500 μL of 1 mg/mL) was added with MnO cores as the fluorescent tag.

### uMUC-1 Peptide Attachment to NEMO Particles

The uMUC-1 tumor-specific peptide EPPT (2-azidoacetic acid-YCAREPPTRTFAYWG) and its scrambled peptide version (2-azidoacetic acid-PRYCGWTEARATPYF) were obtained from AnaSpec (≥95% by HPLC). Click-chemistry was used to form a covalent bond between the terminal azide group on the peptide and the alkyne group on the PLGA–PEG NEMO particles. Briefly, ∼30 mg of NEMO particles or blank particles were mixed with ∼70 μg of ligand (TBTA), ∼25 μg of copper(II) acetate monohydrate, ∼7 pmol of the uMUC-1 targeting peptide or scrambled peptide, and deionized water in a 50 mL Falcon tube. After mixing with a bath sonicator and vortex, the Falcon tube was deoxygenated by bubbling argon for 15 min. To reduce copper(II) to copper(I), 0.1 mL of sodium ascorbate stock solution (5.4 mM) was added to initiate the click chemistry reaction. The solution was mixed overnight on a stir plate under inert conditions. Conjugated particles were washed with deionized water (3 times at 4255 RCF, 2 times at 1734 RCF), aliquoted, and stored at −80 °C.

### Tumor-Targeted NEMO Particle Physical and Chemical Characterization

The morphology of uMUC-1 conjugated NEMO particles (EPPT-NEMO) was obtained via scanning electron microscopy (SEM) with a Hitachi Scanning Electron Microscope at 5 kV. The hydrodynamic particle size was measured using a Nanosight NS300 (Malvern Instruments). Using the Zetasizer Nano ZS (Malvern Instruments), zeta potential (ζ-potential) was measured of conjugated nanoparticles suspended in deionized water; the hydrodynamic size of the particles before and after a 24 h incubation in PBS or RPMI 1640 media supplemented with 10% FBS was measured to assess stability in physiological conditions. Fourier transform infrared spectroscopy (FTIR) of EPPT-NEMO particles evaluated the surface chemistry using a DIGILAB FTS 7000 FTIR spectrometer (PIKE technologies) equipped with a GladiATR attenuated total reflectance module.

### Mn Loading and pH-Dependent NEMO Particle Controlled Release

Mn loading of EPPT-NEMO particles was calculated by digesting ∼2 mg of particles in 150 μL of HCl. After complete digestion, Mn content was analyzed under EPA Method 200.8 Revision 5.4 on an inductively coupled plasma-mass spectrometer (ICP-MS) (PerkinElmer NexION 2000 ICP Mass Spectrometer). Mn^2+^ controlled release at different pH levels over 24 h was performed as previously described.^9^ Briefly, 5 mg of particles were suspended in 1 mL of PBS pH 7.4 (physiological pH), 20 mM citrate buffer pH 6.5 (tumor microenvironment pH), or 20 mM citrate buffer pH 5 (endosome/lysosome pH). Suspensions were incubated at 37 °C with slow rotation in a hybridization oven. After 1, 2, 4, 8, and 24 h, supernatants were collected at 9391 RCF and assessed for Mn^2+^ release by inductively coupled plasma-optical emission spectrometry (ICP-OES) analysis (Agilent 720 ICP-OES).

### MRI Signal of Intact and Digested NEMO Particles

Samples collected from Mn^2+^ controlled release after 1 h were diluted 100-fold and analyzed for MRI properties on a 1.0 T Bruker ICON MRI. The longitudinal relaxation rate, or *R*_1_, was measured from MRI scans acquired using a RARE sequence (TE = 10.68 ms, TR = 25.6 to 12,800 ms, resolution = 234 μm). Images were evaluated with MicroDicom, and data were fitted to follow the *R*_1_ longitudinal relaxation equation (eq 1 below) with MATLAB:
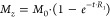
1where *M*_*z*_ is the longitudinal magnetization aligned along the *z*-axis at time *t*, and *M*_0_ is the magnetization at equilibrium.

Additionally, intact particles suspended in 0.5% agarose and HCl-digested particles were imaged at different concentrations of Mn (0 to 2.2 mM for intact particles and 0 to 0.8 mM for digested particles) following the same protocol as above. Data were plotted and fitted to follow eq 2 to calculate the *T*_1_-weighted relaxivity (*r*_1_) properties of the particles:
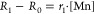
2where *R*_0_ is the relaxation rate when no Mn is present, and [Mn] is the concentration of manganese in mM.

### uMUC-1 Peptide Attachment Characterization

Qualitative characterization of EPPT peptide attachment was performed with X-ray photoelectron spectroscopy (XPS) using a PHI VersaProbe 5000 Scanning X-ray Photoelectron Spectrometer (ULVAC-PHI); XPS evaluated EPPT-conjugated NEMO particles having a higher peptide content for proof-of-concept. High-resolution nitrogen scans were acquired with a pass energy of 23.5 eV and an energy step of 0.1 eV. Quantitative characterization of the attached peptide density to NEMO particles was performed using the Pierce Quantitative Fluorometric Peptide Assay as per the manufacturer’s instructions. The unknown concentration of EPPT peptide present on NEMO particles post-click chemistry versus pre-click chemistry (control) was determined based on the standard curve of *Final Standard Concentration for Individual Peptides* as provided in the manufacturer’s protocol. Hydrodynamic size and particle concentration (Malvern NanoSight NS300) were obtained and eq 3 was used to calculate peptide density:
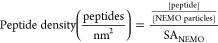
3where [peptide] is the concentration of peptide calculated from the standard curve and Avogadro’s number; [NEMO particles] is the concentration of nanoparticles found from Nanoparticle Tracking Analysis (NanoSight, NS300, Malvern Instruments), and SA_NEMO_ is the NEMO particle surface area calculated from the hydrodynamic size.

### Flow Cytometry of uMUC-1 Cell Expression

Cell suspensions containing 1 × 10^5^ MCF10A or T47D cells were incubated with human IgG on ice for 30 min and then washed twice with PBS. To assess MUC-1 and uMUC-1 expression, cells were incubated with PE-conjugated monoclonal antibodies CD227 (1:100 dilution) or SM3 (1:200 dilution), respectively, on ice for 30 min. For unlabeled cells, no antibody was used. Cells were washed three times with PBS, fixed with 0.4% formaldehyde overnight, and analyzed using a BD LSRFortessa with a 561 nm Sapphire laser.

### In Vitro NEMO Particle Cytotoxicity

Cells were plated and incubated overnight at 37 °C, and fresh media containing different concentrations of EPPT-NEMO particles ([Mn] = 0.81, 1.63, 3.25, 6.50, and 13.00 μg/mL) were added. Cells incubated with media served as negative controls. Cell viability was measured using the WST assay according to the manufacturer’s instructions after 1 and 24 h of exposure.

### MRI Cell Labeling with NEMO Particles

Cells were plated and grown to confluency at 37 °C, and fresh media was added with or without EPPT-NEMO particles or scrambled peptide conjugated NEMO particles at a dose of [Mn] = 6.5 μg/mL; peptide conjugated blank particles were also added as an additional control. After varying exposure times (15, 30, 45, and 60 min), media in contact with cells was collected and cells were washed three times with PBS to remove any additional uninternalized particles. Cells were detached, counted, pelleted in PBS, and assessed for MRI signal at 1.0 T. The collected media was centrifuged to pellet down intact NEMO particles, and the supernatant was analyzed for MRI signal at 1.0 T.

Cell pellets in PBS were imaged coronally, and collected media were imaged axially in 0.65 mL microcentrifuge tubes following similar protocols as above (RARE sequence, TE = 10.68 ms, TR = 25.6 to 6400 ms (cells) and 25.6 to 12,800 ms (media), resolution = 234 μm). Data were fitted using eq 1 to calculate *R*_1_ of the NEMO particle-labeled and unlabeled (control) cells and media. The percent change in relaxation rate (%Δ*R*_1_) for cells and media was calculated using eq 4:
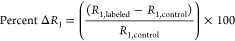
4where *R*_1*,*labeled_ is the longitudinal relaxation rate of cells and media labeled with NEMO particles, and *R*_1*,*control_ is the *R*_1_ of unlabeled cells and media unexposed to particles. After imaging, cell pellets were digested with 150 μL of HCl and analyzed with ICP-MS to obtain the total Mn content per cell.

### Confocal Microscopy of NEMO Particle-Labeled Cells

Cells were plated in 8-well chamber slides and incubated at 37 °C overnight to allow for attachment. Cells were then stained with either Rab5a-RFP to visualize early endosomes, Rab7a-GFP to visualize late endosomes, or Lamp-1-GFP to visualize lysosomes using the CellLight fluorescent protein labeling kit according to the manufacturer’s instructions. Once the organelles were stained, cells were dosed with fresh media containing Cy5-labeled EPPT-NEMO particles or Cy5-labeled scrambled peptide-conjugated NEMO particles (control) at [Mn] = 6.5 μg/mL. After 15, 30, or 45 min of incubation at 37 °C, cells were washed three times with PBS to remove uninternalized NEMO particles before fixing with 4% formaldehyde and staining nuclei with Hoechst 33342 for 20 min at room temperature. Three fields of view were collected per chamber slide (*n* = 3 slides per group) on an inverted Nikon A1R confocal microscope with an oil 40x objective with the following lasers: 405 nm (Hoechst 33342, nuclei), 488 nm (GFP, late endosomes or lysosomes), 555 nm (RFP, early endosomes), and 640 nm (Cy5, NEMO particles).

### Image Analysis

Confocal images were subsequently processed using the FIJI software. After splitting the channels, the brightness/contrast of organelles (RFP or GFP) was adjusted to subtract the background. The adjusted brightness/contrast was then propagated to all channels. After processing the images, colocalization analysis of NEMO particles with endosomes and lysosomes was conducted using FIJI. The channels for organelles (red or green) and NEMO particles (pseudocolored yellow) were merged to form an RGB image. Thresholding was adjusted based on the overlap region between red and yellow for early endosomes or an overlap between green and yellow for late endosomes and lysosomes. The area of NEMO particles colocalized with the respective organelle (CA_NEMO_) and the total area of cells and particles (TA) in the image were obtained. The percent colocalization was calculated based on eq 5:
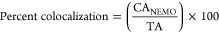
5

### Statistical Analysis

All statistical analyses were performed in GraphPad Prism 10.4.1. Statistical analysis of pH-dependent NEMO particle controlled release was done by performing two-way ANOVA with Holm-Šídák correction. Statistical analysis of the in vitro NEMO cytotoxicity study was performed using one-way ANOVA with Dunnett’s correction against the unlabeled control group. All other statistical analyses were done by performing two-way ANOVA with Tukey’s correction. All experiments were performed in at least triplicate. *p*-values <0.05 were considered significant.

## Results and Discussion

**Figure 1:**
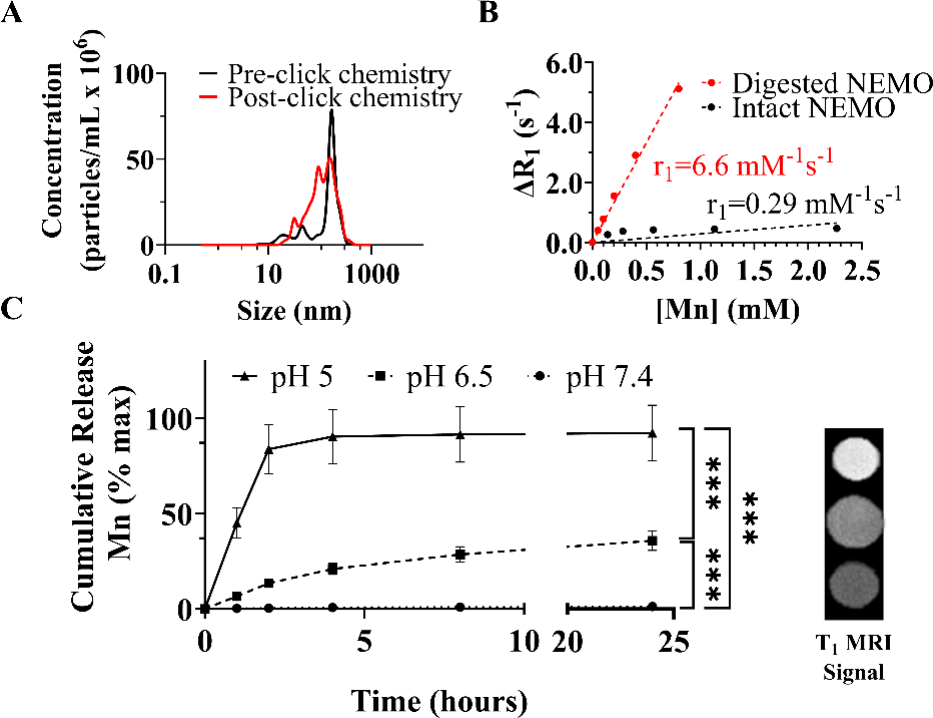
Sizing and MRI properties of EPPT-NEMO particles. (A) Hydrodynamic size of EPPT-NEMO particles pre- (black line) and post (red line) peptide attachment. (B) *T*_1_-weighted *r*_1_ properties of intact EPPT-NEMO particles suspended in 0.5% agarose (black) or digested in HCl (red). (C) Cumulative release of Mn^2+^ from EPPT-NEMO particles over 24 h after incubation at pH 7.4 (dotted line), pH 6.5 (dashed line), and pH 5 (solid line). Note that max Mn^2+^ release occurred rapidly at pH 5 mimicking endosomes/lysosomes to produce the brightest *T*_1_ MRI contrast after 1 h (top circle in the image to the right). Error bars are standard deviation. *** *p* ≤ 0.001.

Synthesized MnO and NEMO particles presented similar characteristics to the work previously presented, where ∼19 nm MnO cores were successfully encapsulated in PLGA–PEG polymer, creating spherical particles of 166 ± 9 nm (Figure 1A).^38,39^ Then, the novel combination of the tumor-targeting peptide, EPPT, against uMUC-1 and pH-responsive NEMO particles was created via click chemistry. During the reaction, the azide-termination on the peptide reacted with the alkyne-termination from the PLGA–PEG shell, forming a triazole bond. EPPT-NEMO particles were characterized through sizing of NEMO pre- and post-peptide attachment, quantification of attached peptide, *T*_1_ MRI properties, Mn loading, controlled release, chemistry, assessment of morphology, and stability (Figures 1 and S1, Table 1).

**Table 1:** Properties of uMUC-1 Targeted NEMO Particles (EPPT-NEMO)

property	value
average mean ± std	166 ± 9 nm
ζ-potential	–18.4 ± 0.6 mV
Mn loading capacity	33%
peptide density	8 × 10^–2^ peptides/nm^2^

XPS was first performed to verify the presence of the peptide qualitatively by observing the increase in nitrogen levels before and after attachment due to the presence of nitrogen in the amino acids that form the peptide (Figure S1D). However, one of the limitations of this technique is the inability to precisely quantify the number of peptide chains per particle or peptide density, as it is not possible to create a standard curve to calculate concentration. For this purpose, the Pierce quantitative fluorometric peptide assay (Thermo Fisher) was used to determine the peptide density attached to NEMO particles, which was measured to be 8 × 10^–2^ peptides/nm^2^. The peptide density achieved was on par with other tumor-targeted nanoparticles conjugated with similar peptide sequences.^24^

For physical and chemical characterization, conjugated particles presented a spherical morphology, size, and ζ-potential that would maximize cell uptake and also facilitate delivery to tumors when transitioned to in vivo studies.^40^ Additionally, the ability of this novel contrast agent to switch from an “OFF” state into an “ON” state was observed at pH 5, as MnO required acidic environments to dissociate into Mn^2+^ and create a robust MRI signal (Figure 1C).^9,10^ This switchability is marked by a ∼23-fold increase in their relaxivity, *r*_1_, transitioning from 0.29 mM^–1^ s^–1^ (intact particles) to 6.6 mM^–1^ s^–1^ (fully digested particles), as shown in Figure 1B. Clinically used Gd-based contrast agents possess an *r*_1_ range of 3.5 to 3.8 mM^–1^ s^–1^.^41^ Therefore, the stronger signal from Mn^2+^ released could assist in detecting smaller tumors.

**Figure 2:**
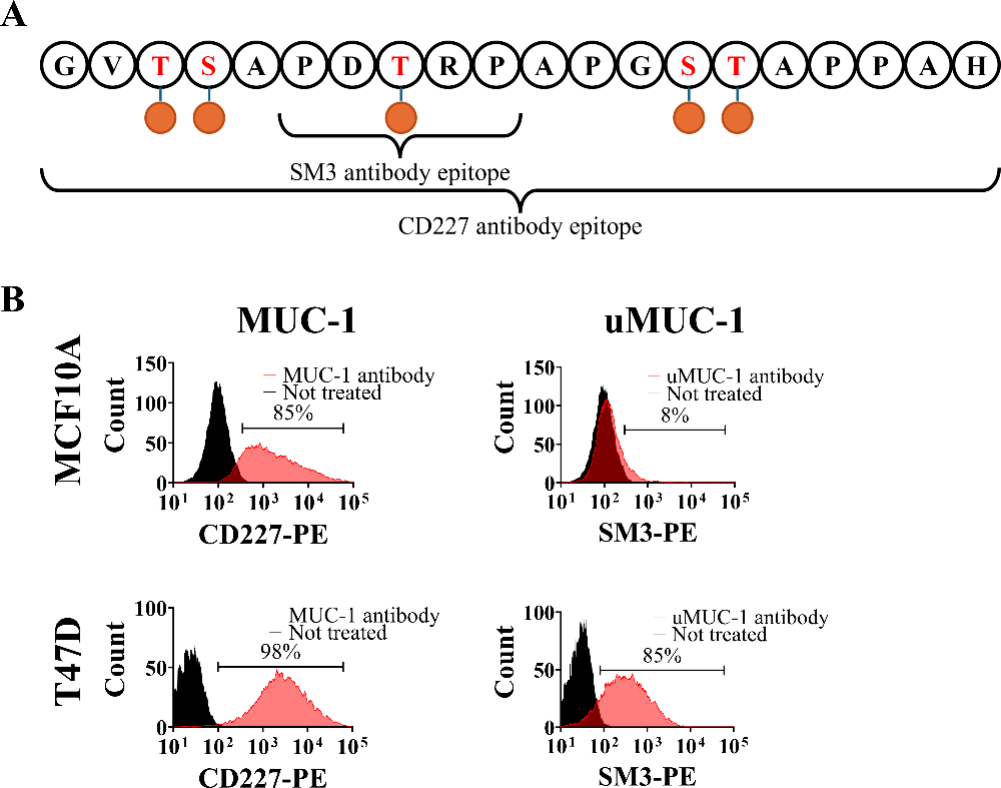
Schematic of MUC-1 glycoprotein and expression of MUC-1 and uMUC-1 on benign versus malignant human mammary cells. (A) VNTR segment of MUC-1 with its O-glycosylations (orange circles). The PDTRP epitope is only visible with underglycosylation, as in breast cancer. (B) Expression of MUC-1 and uMUC-1 on benign MCF10A and malignant T47D cells were analyzed by flow cytometry. Unlabeled cells are colored black; cells labeled with CD227 or SM3 antibodies are represented in red. While the control cell line MCF10A showed a high level of MUC-1 expression, it did not express the tumor biomarker, uMUC-1. Meanwhile, T47D demonstrated high levels of both MUC-1 and uMUC-1 expression.

To test our hypothesis of EPPT-NEMO being more specific toward breast cancer cells (uMUC-1+) than benign mammary cells (uMUC-1-), cellular uptake studies were performed. First, the uMUC-1 expression was confirmed in human benign and mammary carcinoma cell lines. Two different antibodies, CD227 and SM3, that bind to two separate epitopes of the MUC-1 amino acid sequence were used to explore the cellular expression levels of heavily glycosylated MUC-1 (CD227) compared to underglycosylated MUC-1 (SM3) by flow cytometry (Figure 2A). The two human cell lines used were MCF10A, a nontumorigenic epithelial cell line as the control, and T47D, a luminal A breast cancer cell line. Figure 2B shows how the control cell line, MCF10A, expressed high levels of MUC-1 (85%) but minimal levels of uMUC-1 (8%). On the other hand, the breast cancer cell line had high levels of MUC-1 (98%) and uMUC-1 (85%). These results were expected since it has been previously reported that noncancerous cell lines present high levels of O-glycosylation on the variable number of the tandem repeats region, which will allow CD227 antibody binding but block SM3 antibody attachment due to the inaccessibility of PDTRP. In contrast, these sugar chains are lost in cancerous cells to expose the protein backbone, allowing the SM3 antibody to bind specifically to uMUC-1.^25,33,42,43^ As the attached uMUC-1 targeting peptide, EPPT, on the NEMO particles binds to the same epitope as the SM3 antibody, it was hypothesized that the EPPT conjugated particles would enable differentiation between noncancerous versus cancerous cell lines due to their distinct uMUC-1 expression levels and promote subsequent differential NEMO particle uptake and MRI signal generation.

**Figure 3:**
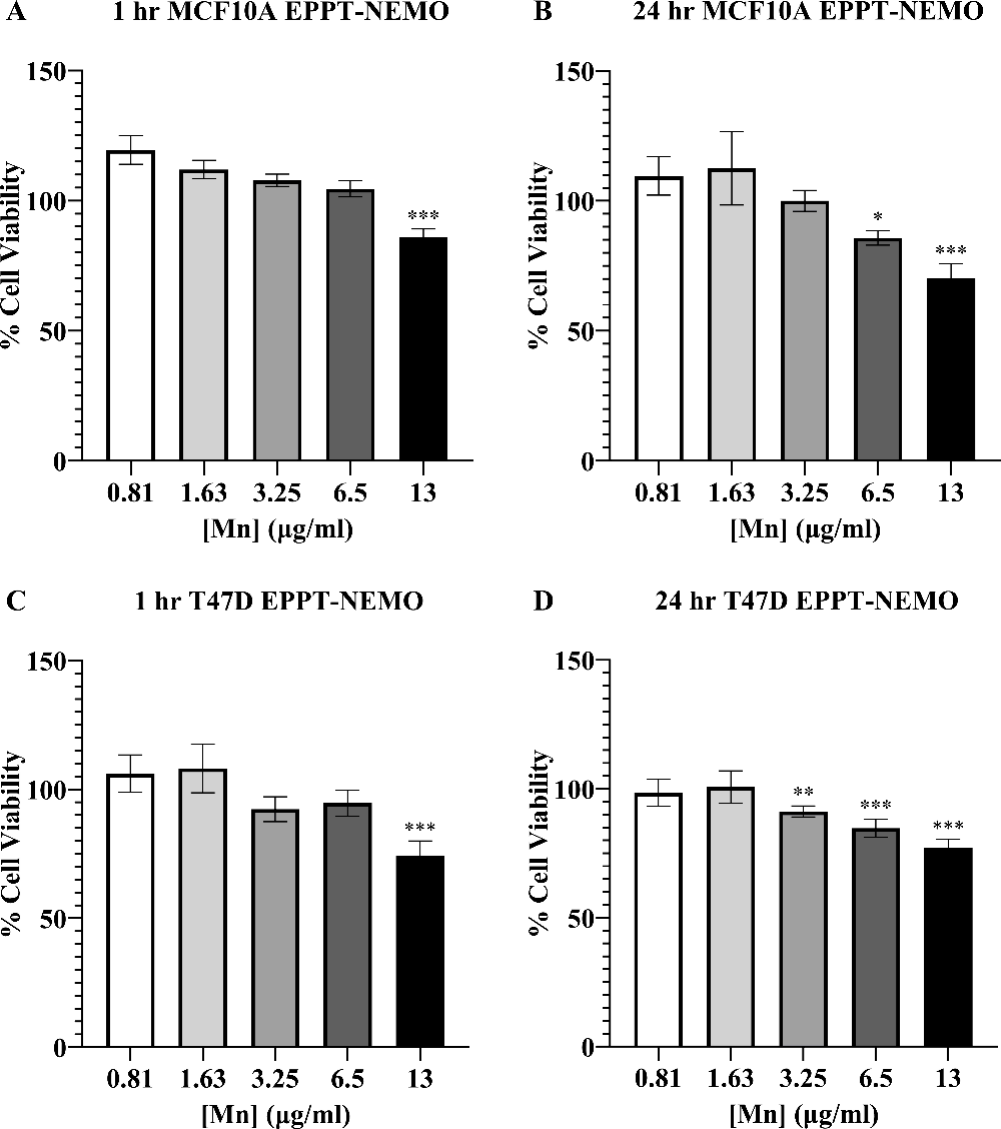
In vitro viability of human mammary cells exposed to EPPT-NEMO particles for 1 and 24 h. WST assay results of EPPT-NEMO particle exposure to (A) MCF10A for 1 h, (B) MCF10A for 24 h, (C) T47D for 1 h, and (D) T47D for 24 h. The maximum cytotoxicity was detected at the highest dose [Mn] = 13.00 μg/mL for both the control and tumorigenic cell line. Cell viability for all Mn doses were normalized to control (cells labeled with media). Statistics are shown for any significant reductions in viability between each Mn dose and the unlabeled control. Error bars are standard deviation. * *p* ≤ 0.05, ** *p* ≤ 0.01, *** *p* ≤ 0.001.

The cytotoxicity of EPPT-NEMO particles was assessed by the WST assay (Figure 3) after 1 and 24 h to determine the safest dose for cellular uptake studies. Cell viability was evaluated at 1 h to determine any toxicity associated with the cell labeling times used for MRI and confocal studies. As particle concentration increased, cell viability decreased to a minimum of ∼75% at the highest dose of [Mn] = 13.00 μg/mL in both cell lines at 1 and 24 h. From [Mn] = 0.81 to 6.5 μg/mL, cells exposed to EPPT-NEMO had a high viability of >85%. Based on this data, the highest safe dose of [Mn] = 6.5 μg/mL was chosen to maximize the MRI signal for the cellular uptake studies.

**Figure 4:**
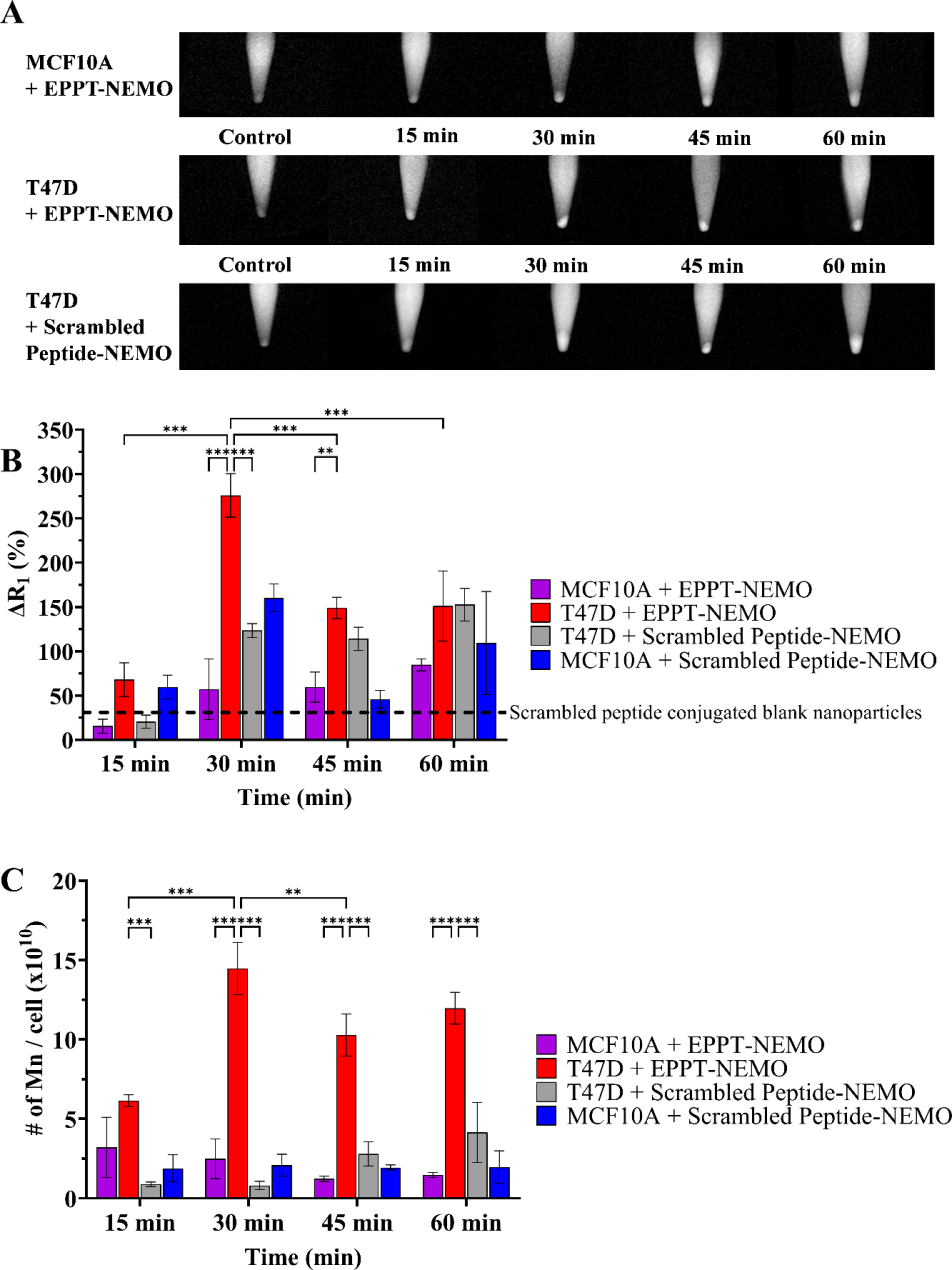
*T*_1_-weighted MRI scans, quantification of MRI signal change, and Mn content of benign and malignant mammary cells labeled with NEMO particles over 60 min. (A) Representative *T*_1_-weighted MRI scans of MCF10A and T47D cell pellets labeled with media only (control) or [Mn] = 6.5 μg/mL of NEMO particles over 60 min. (B) Quantification of change in relaxation rate Δ*R*_1_ for MCF10A versus T47D cells. The enhancement in *T*_1_-weighted MRI signal of T47D cells labeled with EPPT-NEMO reached its peak at 30 min and then decreased. From 30 to 45 min, %Δ*R*_1_ was significantly greater for T47D cells labeled with EPPT-NEMO compared with MCF10A cells. MRI signal enhancement of scrambled peptide conjugated blank particles was demonstrated to be ∼33% (black dotted line), raising the baseline MRI signal of scrambled peptide groups. (C) ICP-MS quantification of Mn cell content. T47D cells labeled with EPPT-NEMO had significantly greater internalized Mn compared to both control groups from 30 to 60 min. Error bars are standard deviation. ** *p* ≤ 0.01, *** *p* ≤ 0.001.

Cell uptake studies were conducted where the cells were exposed to [Mn] = 6.5 μg/mL EPPT-NEMO or scrambled peptide-NEMO to determine the MRI signal of the labeled cells and media over 60 min. We hypothesized that the uMUC-1 targeting of the EPPT-NEMO particles would facilitate increased cell uptake, intracellular MnO dissolution to Mn^2+^, and a higher MRI signal over 60 min for the malignant T47D cells. The control groups, i.e., MCF10A benign cells with EPPT-NEMO or MCF10A cells and T47D breast cancer cells with scrambled peptide-NEMO, would have lower MRI signal due to the lack of uMUC-1 cellular expression or lack of EPPT nanoparticle targeting, respectively. As shown in Figure 4A,B (see Tables S1 and S2 for numerical values), the MRI signal of EPPT-NEMO in T47D cells significantly peaked to ∼276% signal enhancement at 30 min, which was significantly greater than that of MCF10A cells labeled with EPPT-NEMO (∼57%), T47D cells labeled with scrambled-peptide NEMO (∼123%), and MCF10A cells labeled with scrambled-peptide NEMO (∼160%). At 45 min, the MRI signal change (%Δ*R*_1_) decreased to ∼150% enhancement for T47D cells + EPPT-NEMO; however, the MRI signal remained significantly higher than control MCF10A cells labeled with EPPT-NEMO. To our surprise, the MRI signal of T47D cells incubated with scrambled-peptide-NEMO increased by ∼6.5-fold by 30 min and plateaued for the rest of the time points. By 60 min, the change in MRI signal in T47D cells labeled with EPPT-NEMO and scrambled peptide NEMO was nearly identical.

To gain further insight into the MRI trends, the Mn content of the labeled cells was measured with ICP-MS (Figure 4C). Similar to the pattern observed on MRI, T47D cells labeled with EPPT-NEMO had the highest intracellular Mn content at 30 min, which significantly decreased at 45 min, indicating some exocytosis of the internalized particles or released Mn^2+^ ions. The intracellular Mn content of the control groups (MCF10A cells + EPPT-NEMO, T47D cells + scrambled-peptide NEMO, and MCF10A cells + scrambled-peptide NEMO) was significantly lower than T47D cells labeled with EPPT-NEMO from 30 to 60 min, demonstrating the specificity of the uMUC-1 peptide targeting.

By comparing the MRI signal versus Mn cellular content (Figure 4B,C), it is evident that the higher Mn content in T47D cells labeled with EPPT-NEMO does not necessarily translate to a higher MRI signal at later time points. Based on the ICP-MS results, Mn cellular content remains high in T47D cells labeled with EPPT-NEMO from 30 to 60 min, but the observed *T*_1_ MRI signal enhancement markedly decreased for 45 and 60 min post-labeling. We hypothesize that this reduction in %Δ*R*_1_ can be attributed to some quenching of Mn^2+^ due to higher conversion of MnO to Mn^2+^ at later time points, coupled with the confined endosomal space limiting water relaxation and the increased *T*_2_ shortening effects with higher Mn^2+^ concentrations, which is consistent with published literature.^44−46^

The %Δ*R*_1_ of MCF10A cells and T47D cells labeled with scrambled peptide-NEMO (Figure 4B) was also as high as 160% despite the low Mn intracellular content (Figure 4C). The observed increase in MRI signal enhancement in scrambled peptide controls compared to those in EPPT-NEMO particles may be attributed to differential quenching of Mn^2+^. As stated above, the EPPT peptide demonstrates a higher uptake of NEMO particles in malignant T47D cells, leading to an increased conversion of MnO to Mn^2+^, which is hypothesized to result in *T*_1_ MRI signal quenching at later time points. Since cells labeled by scrambled peptide NEMO did endocytose low levels of Mn according to ICP-MS, they would experience some *T*_1_ MRI signal enhancement without quenching.

Alternatively, it may be possible that the scrambled peptide activates certain signaling pathways, which results in a higher MRI signal, even though the uptake is low in both T47D and MCF10A cells (Figure 4C). To mitigate the ambiguity between uptake and MRI signal enhancement of scrambled peptide-NEMO particles, cell labeling of T47D and MCF10A cells with both EPPT- and scrambled peptide-conjugated blank particles with no MnO inside was carried out at 30, 45, and 60 min. The data demonstrated ∼33% MRI signal enhancement for scrambled peptide-conjugated blank particles compared to no signal enhancement when cells were labeled with EPPT-conjugated blank particles. This raises the baseline signal for scrambled-peptide groups. In other words, scrambled peptide particles without metal were able to enhance the MRI signal, which partially explains why a higher MRI signal is observed for scrambled peptide-NEMO particles despite low cell uptake by ICP-MS. We also evaluated the zeta potential of NEMO particles and blank particles with attached EPPT peptides versus scrambled peptides to rule out any differential impact of electrostatic surface charge. Peptide type did not significantly change zeta potential as shown by the obtained values of −18.4 ± 0.6 mV for EPPT-NEMO versus −16 ± 0.7 mV for scrambled peptide-NEMO and −26.6 ± 0.6 mV for EPPT-blank nanoparticles versus −31 ± 0.4 mV for scrambled peptide-blank nanoparticles. This new discovery regarding scrambled-peptide-induced *T*_1_-weighted MRI enhancement warrants further investigation.

Additionally, the MRI scans of the media were acquired at all time points to analyze the percent signal change of exocytosed Mn^2+^ ions when compared to unlabeled cells (Figure S2). It is important to observe that MRI signal alterations remained below 100% across all experimental groups. The percentage change in longitudinal relaxation rate (%Δ*R*_1_) in the media surrounding T47D cells treated with EPPT-NEMO showed an upward trend from 15 to 45 min. When T47D and MCF10A cells labeled with EPPT-NEMO were compared, no notable disparities in %Δ*R*_1_ were detected in their respective media between 15 and 45 min. Surprisingly, T47D cells treated with the scrambled peptide-NEMO exhibited significantly higher %Δ*R*_1_ values at both 15 and 30 min, in contrast to their counterparts incubated with EPPT-NEMO.

The MRI signal of NEMO particles is elicited when they are endocytosed into the cells inside early endosomes (pH = 6.5), late endosomes (pH = 5.5), and lysosomes (pH = 4.5), having a range of low pH^47,48^ to dissociate MnO into Mn^2+^ and O^2–^. The endocytic pathway consists of vesicles known as endosomes, which progress linearly from an early endosome to a late endosome before ultimately fusing with cellular compartments, lysosomes. Lysosomes are repositories of specialized catalytic proteins that facilitate degradation by having the most acidic pH. Any material engulfed by the cell through this internalization pathway is first sequestered within endosomes and, subsequently, delivered to lysosomes, where it undergoes active disintegration by enzymatic processes.

**Figure 5:**
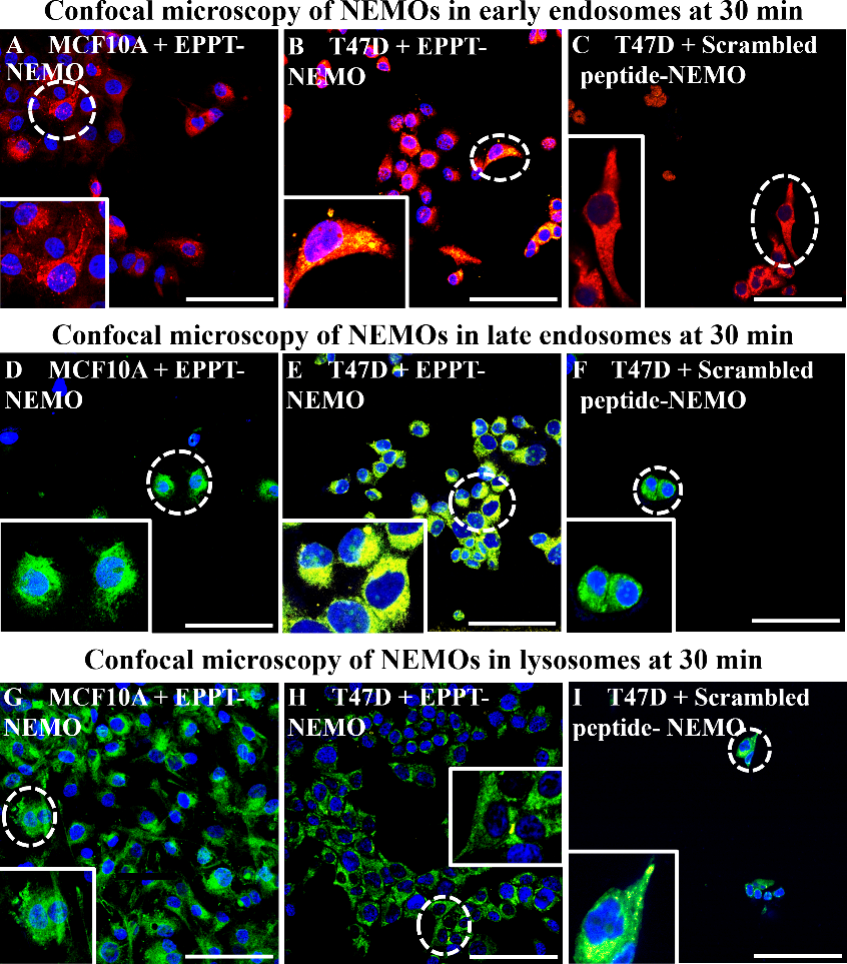
Representative confocal microscopy images of NEMO particles (pseudocolored yellow) colocalized with early endosomes (red, A–C), late endosomes (green, D–F) and lysosomes (green, G–I) of MCF10A and T47D cells at 30 min. Representative cells (dotted circles) are shown at a higher magnification in the boxed insets. EPPT-NEMO particles are seen to colocalize with early and late endosomes after 30 min. Scale bars: 100 μm.

To understand how intracellular trafficking of NEMO particles within the vesicles affects the MRI signal, we performed cell uptake studies and labeling with conjugated NEMO particles to assess the localization of particles within early and late endosomes versus those within lysosomes. T47D and MCF10A cells were exposed to either EPPT-NEMO or scrambled peptide-NEMO particles over 45 min at a concentration of [Mn] = 6.5 μg/mL, similar to that in MRI studies. After incubating NEMO particles with cells at desired time points, cells were fixed and imaged under a confocal microscope to determine colocalization of Cy5 NEMO particles (pseudocolored yellow) with early endosomes (RFP), late endosomes (GFP), and lysosomes (GFP) as shown in Figure 5. We hypothesized that the MRI signal would be greater when the NEMO particles were localized in late endosomes and/or lysosomes due to their pH being more acidic compared to early endosomes. This hypothesis is based on enhanced dissociation of MnO to Mn^2+^ at a lower pH.

**Figure 6:**
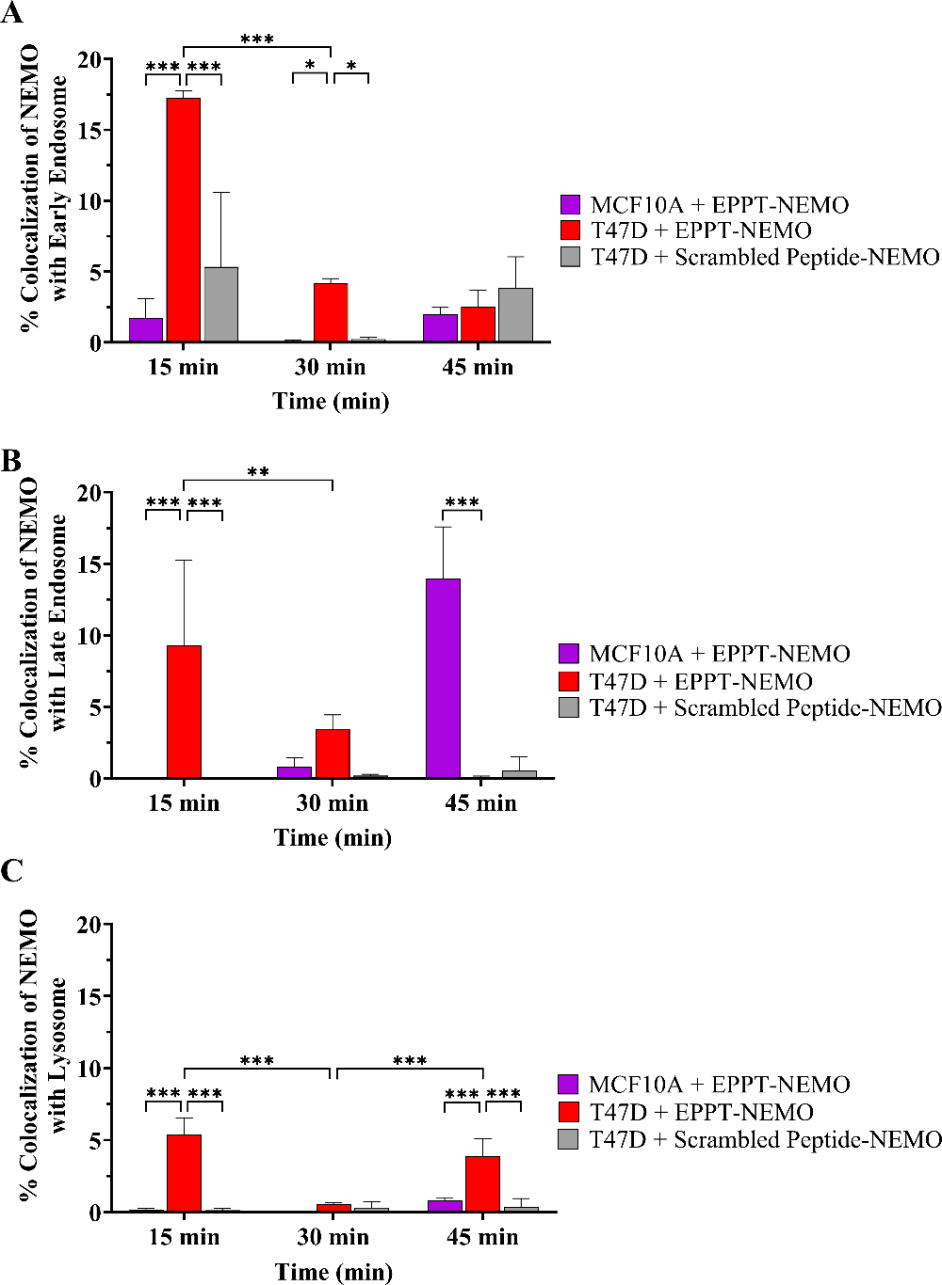
Quantification of the colocalized area of NEMO particles with endosomes and lysosomes in benign versus malignant mammary cells over 45 min. Percent colocalization of EPPT-NEMO particles and scrambled peptide-NEMO particles with (A) early endosomes (B) late endosomes and (C) lysosomes in MCF10A and T47D cells over time. Error bars are standard deviation. * *p* ≤ 0.05, ** *p* ≤ 0.01, *** *p* ≤ 0.001.

Since the peak of the MRI signal was observed starting at 30 min in breast cancer cells, we were interested in determining the percentage of NEMO particles colocalized with endosomes and lysosomes at time points until 45 min to determine particle uptake (Figure 6). We observed the highest colocalization of EPPT-NEMO with early endosomes, late endosomes, and lysosomes of T47D compared to scrambled peptide-NEMO with T47D and MCF10A with EPPT-NEMO after 15 min. This could indicate that at 15 min, the EPPT-NEMO gets taken up by T47D but does not degrade enough to have a strong MRI signal enhancement (Figure 4B). Interestingly, we observed overall low colocalization of EPPT-NEMO at 30 and 45 min with all three early endosomes, late endosomes, and lysosomes in T47D cells. This can be attributed to significant digestion of the particles in the low pH endosomes/lysosomes from 15 to 45 min, which would dissociate the polymers and release the encapsulated Cy5 dye to diminish its detectability on confocal microscopy. In summary, confocal microscopy confirmed EPPT-NEMO particles are taken up by the endosomal/lysosomal pathway in cancer cells to facilitate their degradation and activation of the MRI signal.

MnO nanoparticles have demonstrated significant potential as *T*_1_ MRI contrast agents, with their efficacy varying across studies depending on nanoparticle design, surface modification, and cellular targeting. Comparing the reported *T*_1_ relaxation times and *R*_1_ values from previous studies with our data set reveals significant differences in performance. For instance, at our low labeling dose of [Mn] = 6.5 μg/mL or 0.12 mM Mn, we achieved a signal enhancement of 276% within a clinically relevant time of 30 min in the malignant cells incubated with EPPT-NEMO. Notably, other studies have shown lower MRI signal enhancement in malignant cells labeled with higher Mn doses over longer incubation times. For example, Li et al. achieved a signal enhancement of ∼114% in renal carcinoma cells after 24 h using a dose of 0.2 mM Mn delivered by an AS114 aptamer-targeted MnO-PEG nanoprobe.^49^ Although their MnO-PEG nanoparticles displayed higher *r*_1_ than our EPPT-NEMO particles outside of cells (12.9 mM^–1^ s^–1^ for their intact particles versus 6.6 mM^–1^ s^–1^ for our fully digested particles), this high baseline signal of their intact particles would limit pH responsiveness and required 24 h for the control untargeted probe to clear out of mouse tumors in vivo for *T*_1_-weighted MRI signal to return to precontrast levels.^49^ Due to their specific intracellular activation in malignant cells, we hypothesize that NEMO particles should also demonstrate faster in vivo benign versus malignant tumor discrimination without the need for clearance of unendocytosed intratumoral probes. Furthermore, Hu et al. demonstrated a signal enhancement in lung carcinoma cells at a dose of [Mn] = 100 μg /mL after incubating for 6 h^50^; however, the MRI scans were not quantified for % signal enhancement. In addition, the lack of active targeting on their MnO nanoparticles may limit the differentiation of benign from malignant tumor cells. Though it is difficult to directly compare the results due to multiple factors such as the different nanoparticle designs, cell types, cell receptor targets, and MRI magnetic field strengths used, our EPPT-NEMO particles show promise as an alternative MRI contrast agent with quick, robust, and specific *T*_1_-weighted signal activation.

There are several limitations associated with our study that can be addressed with future work: (1) Confocal microscopy tracks the Cy5 encapsulated dye inside NEMO particles and not Mn directly. In future studies, X-ray fluorescence microscopy could help to track the elemental Mn^2+^ ions in biological samples over time at cellular levels.^51,52^ (2) As only luminal A T47D breast cancer cells were tested here, evaluating NEMO particle labeling with other breast cancer subtypes including luminal B, HER2, and triple negative would be useful to determine the generalizability of uMUC-1 targeting. (3) As an alternative to the discrete time points for labeling used in this study, live cell imaging could provide further insights into the dynamics of NEMO particle uptake, localization, and dissolution in low pH endosomes and lysosomes. (4) Although endocytosis pathways were not considered herein, additional studies could be performed with different pathway inhibitors during cell labeling to determine NEMO’s uptake mechanism. (5) The in vitro specific *T*_1_ MRI signal enhancement observed provides feasibility for applying NEMO particles for enhanced breast cancer detection. However, in vivo investigation is necessary in preclinical models for future work, which will determine EPPT-NEMO biodistribution, toxicity, and MRI signal activation in breast cancer mouse models versus clinically used Gd-chelates. In order for NEMO particles to be safely used in future clinical applications, a dose response will be performed to determine the lowest effective dose for a specific elevated *T*_1_ MRI signal.

## Conclusions

Novel uMUC-1 targeted NEMO particles were synthesized, characterized, and evaluated for cell uptake and MRI properties in benign versus malignant mammary cells. The uMUC-1 targeting peptide EPPT was successfully attached and on par with other previously reported particles. In vitro studies confirmed increased specificity of EPPT-NEMO with malignant mammary cells when compared to benign mammary cells, with robust *T*_1_-weighted MRI signal generation in breast cancer cells at a clinically relevant time frame of 30 min. Despite the 276% signal enhancement on MRI at 30 min, EPPT-NEMO did not have significant colocalization with the low pH late endosomes and lysosomes in breast cancer cells, which suggested rapid particle degradation, release of encapsulated Cy5, and dilution of its signal on confocal microscopy. Our results demonstrate the feasibility of EPPT-NEMO particles in differentiating malignant from benign mammary cells on *T*_1_-weighted MRI and provide rationale for future in vivo preclinical testing to establish biodistribution, toxicity, and MRI signal activation in mammary tumors.

## Supporting Information Available

Surface chemistry, morphology, stability, and peptide attachment of EPPT-NEMO particles; absolute and relative MRI quantification results of T47D cells incubated with or without contrast agents over 1 h; absolute and relative MRI quantification results of MCF10A cells incubated with or without contrast agents over 1 h; quantification of the MRI signal change of media supernatant collected after exposing cells to NEMO particles over 60 min (PDF)

## Abbreviations

DLS: dynamic light scattering
EPPT: uMUC-1 targeting peptide
EPPT-NEMO: uMUC-1 targeting peptide conjugated NEMO particles
ER: estrogen receptor
FBS: fetal bovine serum
FTIR: Fourier transform infrared spectroscopy
Gd: gadolinium
MnO: manganese oxide
HCl: hydrochloric acid
HER2: human epidermal growth factor receptor 2
ICP-MS: inductively coupled plasma–mass spectrometry
ICP-OES: inductively coupled plasma–optical emission spectrometry
MEGM: mammary epithelial cell growth medium
Mn(II)(acac)_2_: manganese(II) acetylacetonate
MRI: magnetic resonance imaging
MUC-1: Mucin-1
NEMO: nano-encapsulated manganese oxide
PBS: phosphate buffered saline
PLGA: poly(D,l-lactide-*co*-glycolide)
PR: progesterone receptor
PVA: poly(vinyl alcohol)
SEM: scanning electron microscopy
TBTA: tris((1-benzyl)-4-triazolyl)methyl) amine
uMUC-1: underglycosylated mucin-1
XPS: X-ray photoelectron spectroscopy
ζ-potential: zeta potential
