# Supplementary material for: Development of uMUC-1 Targeted NEMO Particles with pH-Activatable MRI Signals for Enhanced Detection of Malignant Breast Cancer Cells
Source: ACS Appl Bio Mater. 2025 May 1;8(5):4251–61. doi: 10.1021/acsabm.5c00365 (PMC12093376; doi:10.1021/acsabm.5c00365)
Supplement: Supplementary file 1 — mt5c00365_si_001.pdf [file mt5c00365_si_001.pdf]

SUPPORTING INFORMATION

Development of uMUC-1 targeted NEMO particles with pH-activatable MRI signal for enhanced detection of malignant breast cancer cells

Dhruvi M. Panchal [a], Alexia R. Gorman [a], Celia Martinez de la Torre [† a], Barrick M. Silverman [a], Anthony J. Scalzo [b], Hunter T. Snoderly [a], Benoit Driesschaert [c, d, e], and Margaret F. Bennewitz [a]*

a. Department of Chemical and Biomedical Engineering, Benjamin M. Statler College of Engineering and Mineral Resources, West Virginia University, Morgantown, WV 26506, USA

b. Department of Biology, Eberly College of Arts and Sciences, West Virginia University, Morgantown, WV, 26506, USA

c. Department of Pharmaceutical Sciences, School of Pharmacy, West Virginia University, Morgantown, WV, 26506, USA

d. In Vivo Multifunctional Magnetic Resonance Center, School of Medicine, West Virginia University, Morgantown, WV, 26506, USA

e. Eugene Bennett Department of Chemistry, Eberly College of Arts and Sciences, West Virginia University, Morgantown, WV, 26506, USA

Corresponding Author

*Margaret F. Bennewitz: Department of Chemical and Biomedical Engineering, Benjamin M. Statler College of Engineering and Mineral Resources, West Virginia University, Morgantown, WV 26506, USA. Email: margaret.bennewitz@mail.wvu.edu.

Present Addresses

[†]Celia Martinez de la Torre: Department of Radiology, Sloan Kettering Institute, Memorial Sloan Kettering Cancer Center, New York, NY, USA, and Molecular Pharmacology Program, Sloan Kettering Institute, Memorial Sloan Kettering Cancer Center, New York, NY, USA

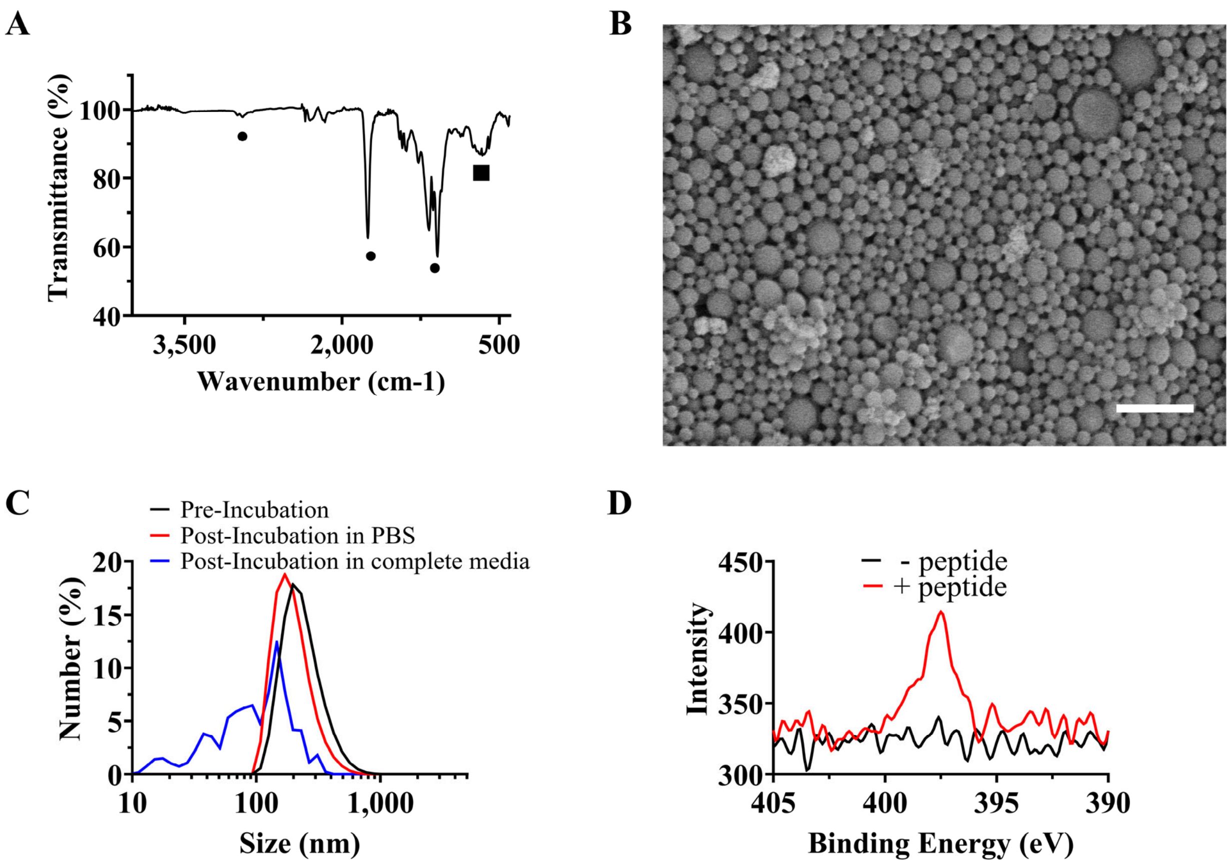

Figure S1. Surface chemistry, morphology, stability and peptide attachment of EPPT-NEMO particles. (A) FTIR spectrum of NEMO particles post-click chemistry with the uMUC-1 targeting peptide EPPT. Both characteristic PLGA peaks (●) and MnO (■) are present in the spectra[1–4]. (B) SEM image of EPPT-NEMO particles showing the spherical morphology of the particles. The scale bar is 1 µm. (C) Hydrated size distribution of EPPT-NEMO particles before (black line) and after 24 hr of incubation in PBS (red line) or RPMI 1640 media supplemented with 10% fetal bovine serum (blue line). Note that the size distribution stayed consistent in PBS and no aggregation or increase in size was noticed post-incubation in complete media, showing the stability of the particles. (D) XPS spectra of the N1s region of the NEMO particles pre- (black line) and post- (red line) click chemistry confirming the peptide attachment.

Table S1. Absolute and relative MRI quantification results of T47D cells incubated with or without contrast agents over 1 hr*

Timepoint	Sample	T_1 (ms)	R_1 (s^{-1})	ΔR_1 (%)
15 min	Control	1083 ± 145	0.93 ± 0.1	-
	EPPT-NEMO	642 ± 67	1.57 ± 0.2	68 ± 19
	Scrambled Peptide-NEMO	890 ± 54	1.13 ± 0.1	21 ± 7
30 min	Control	1370 ± 302	0.75 ± 0.2	-
	EPPT-NEMO	355 ± 24	2.83 ± 0.2	276 ± 25
	Scrambled Peptide-NEMO	596 ± 21	1.68 ± 0.1	123 ± 8
45 min	Control	1192 ± 51	0.84 ± 0.04	-
	EPPT-NEMO	479 ± 23	2.09 ± 0.1	149 ± 12
	Scrambled Peptide-NEMO	558 ± 35	1.8 ± 0.1	114 ± 13
60 min	Control	1177 ± 124	0.86 ± 0.1	-
	EPPT-NEMO	474 ± 80	2.15 ± 0.3	151 ± 40
	Scrambled Peptide-NEMO	464 ± 32	2.16 ± 0.2	153 ± 19
Scrambled peptide conjugated Blank nanoparticles		904 ± 91	1.11 ± 0.1	36 ± 6

Table S2. Absolute and relative MRI quantification results of MCF10A cells incubated with or without contrast agents over 1 hr*

Timepoint	Sample	T_1 (ms)	R_1 (s^{-1})	ΔR_1 (%)
15 min	Control	1142 ± 246	0.9 ± 0.2	-
	EPPT-NEMO	963 ± 64	1.04 ± 0.1	15 ± 8
	Scrambled Peptide-NEMO	698 ± 59	1.44 ± 0.1	60 ± 13
30 min	Control	1626 ± 188	0.62 ± 0.1	-
	EPPT-NEMO	1055 ± 204	0.98 ± 0.2	57 ± 34
	Scrambled Peptide-NEMO	620 ± 36	1.62 ± 0.1	160 ± 16
45 min	Control	1107 ± 202	0.92 ± 0.2	-
	EPPT-NEMO	685 ± 76	1.47 ± 0.2	59 ± 17
	Scrambled Peptide-NEMO	745 ± 52	1.35 ± 0.1	46 ± 10
60 min	Control	1489 ± 92.5	0.85 ± 0.5	-
	EPPT-NEMO	637 ± 24	1.57 ± 0.1	85 ± 7
	Scrambled Peptide-NEMO	594 ± 182	1.78 ± 0.5	109 ± 58
Scrambled peptide conjugated Blank nanoparticles		981 ± 171	1.04 ± 0.2	31 ± 10

**For both Table S1 and Table S2, mean ± standard deviation values are shown.*

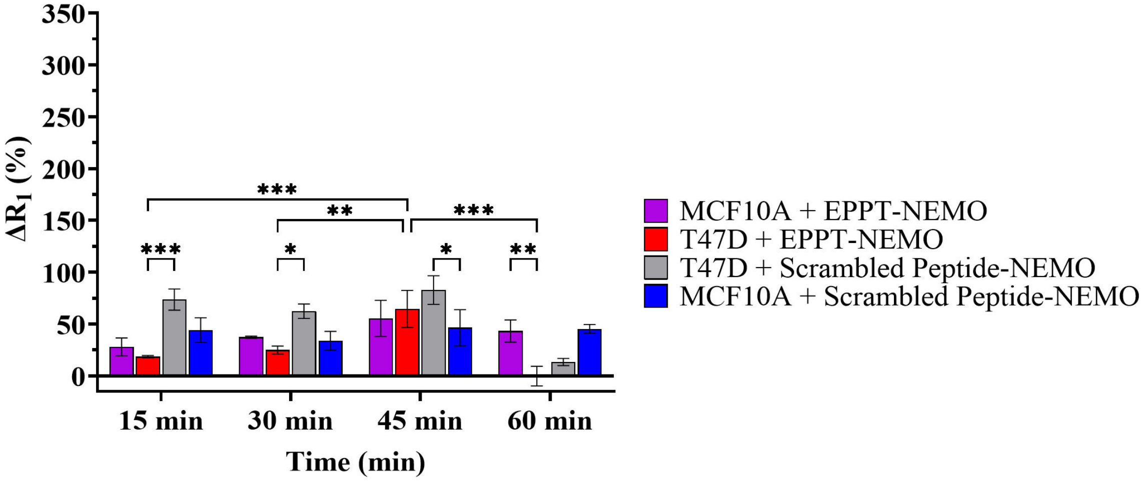

Figure S2. Quantification of MRI signal change of media supernatant collected after exposing cells to NEMO particles over 60 min. Note that MRI signal changes remained <100% for all groups, with %ΔR_1 increasing from 15 min to 45 min in the media for T47D cells incubated with EPPT-NEMO. There was no significant difference in %ΔR_1 in the media between T47D and MCF10A cells incubated with EPPT-NEMO from 15 to 45 min. Interestingly, T47D cells incubated with scrambled peptide-NEMO had significantly higher %ΔR_1 in the media at 15 min and 30 min compared to T47D cells incubated with EPPT-NEMO. The media of MCF10A cells incubated with scrambled peptide-NEMO had similar %ΔR_1 to T47D cells incubated with scrambled peptide-NEMO at all time points except for 45 min. Error bars are standard deviation. * $p \leq 0.05$, ** $p \leq 0.01$, *** $p \leq 0.001$.

REFERENCES

(1) Wang, H.; Zhao, Y.; Wu, Y.; Hu, Y.; Nan, K.; Nie, G.; Chen, H. Enhanced Anti-Tumor Efficacy by Co-Delivery of Doxorubicin and Paclitaxel with Amphiphilic Methoxy PEG-PLGA Copolymer Nanoparticles. *Biomaterials* **2011**, *32* (32), 8281–8290. https://doi.org/10.1016/j.biomaterials.2011.07.032.

(2) Arasoglu, T.; Derman, S.; Mansuroglu, B. Comparative Evaluation of Antibacterial Activity of Caffeic Acid Phenethyl Ester and PLGA Nanoparticle Formulation by Different Methods. *Nanotechnology* **2016**, *27* (2), 025103. https://doi.org/10.1088/0957-4484/27/2/025103.

(3) Jiang, P.; Yu, D.; Zhang, W.; Mao, Z.; Gao, C. Influence of Bovine Serum Albumin Coated Poly(Lactic-Co-Glycolic Acid) Particles on Differentiation of Mesenchymal Stem Cells. *RSC Adv.* **2015**, *5* (51), 40924–40931. https://doi.org/10.1039/C5RA07219K.

(4) Snoderly, H. T.; Freshwater, K. A.; Martinez de la Torre, C.; Panchal, D. M.; Vito, J. N.; Bennewitz, M. F. PEGylation of Metal Oxide Nanoparticles Modulates Neutrophil Extracellular Trap Formation. *Biosensors* **2022**, *12* (2), 123. https://doi.org/10.3390/bios12020123.
